# Physical Fitness and Psychosocial Profiles of Policewomen from Professional Training Courses and Bodyguard Special Police Sub-Unit

**DOI:** 10.3390/ejihpe13090136

**Published:** 2023-09-15

**Authors:** Mariana Carrilho, Vanessa Santos, André Rasteiro, Luís Miguel Massuça

**Affiliations:** 1 Higher Institute of Police Sciences and Internal Security, 1300-663 Lisbon, Portugal; 2 ICPOL, Higher Institute of Police Sciences and Internal Security, 1300-663 Lisbon, Portugal; 3 Exercise and Health Laboratory, CIPER, Faculdade de Motricidade Humana, Universidade de Lisboa, 1495-751 Cruz Quebrada, Portugal; 4 KinesioLab, Research Unit in Human Movement Analysis, Instituto Piaget, 2805-059 Almada, Portugal; 5 First Responder Research Laboratory, University of Kentucky, Lexington, KY 40506, USA; 6 CIDEFES, Lusófona University, 1749-024 Lisbon, Portugal; 7 CIFI2D, Faculty of Sport, University of Porto, 4200-450 Porto, Portugal

**Keywords:** fitness, morphology, perseverance in effort, police, training course, woman

## Abstract

Police work demands a high level of physical fitness (PF) and psychosocial well-being (PSWB) to cope with the challenges and demands of the profession. The profession has historically been male-dominated, and female police officers (POs) face unique challenges and stereotypes. This study aims (i) to identify the PF and PSWB attributes that significantly distinguish the women from three different professional training courses (agents, chiefs, and officers) and (ii) to assess the significance of PF and PSWB attributes on the likelihood that women in professional training courses belonging to a special bodyguard police sub-unit. A cross-sectional analytical study was conducted, involving 102 female POs (professional PO training courses, n = 94; special bodyguard police sub-unit, n = 8). PF (morphology; fitness, including endurance, strength, and flexibility tests) and PSWB (measured through scales for grit and dispositional resilience) attributes were assessed. Significant differences were observed in age, morphological (height and waist-to-hip ratio), fitness (horizontal jump and endurance tests), and PSWB (perseverance in effort and alienation) attributes among the three professional training courses. Stepwise discriminant analysis revealed that waist-to-hip ratio, waist-to-height ratio, relative fat mass, relative muscle mass, horizontal jump, and endurance on exertion significantly distinguished between the three groups. Complementarily, it was observed that the waist-to-hip ratio and sit-up attributes influence the probability of women from the professional training courses joining the special bodyguard police sub-unit. The study highlights that (i) female POs in different professional training courses showed distinct PF and PSWB profiles, and (ii) only two PF attributes (waist-to-hip ratio and sit-up) were predictors for the special bodyguard police sub-unit. In accordance, these findings provide valuable insights for designing training programs to support female POs in improving their PF, psychological well-being, and overall performance in the police profession.

## 1. Introduction

Police work is a profession full of daily challenges and surprises. For police officers (POs), each day is unique and presents new situations and demands, ranging from sedentary moments to extreme physical tasks, such as carrying heavy loads, pursuing suspects, and overcoming obstacles. Maintaining physical fitness (PF) is essential to efficiently face the demands of this profession, as it is considered critical to adequately perform the activities associated with their role [1]. In this context, the literature highlights body composition, cardiorespiratory fitness, strength, muscular endurance, flexibility, agility, and speed as relevant attributes for the performance of the police profession [2,3,4,5], as well as a guarantee of health and well-being [6,7].

In addition to PF (i.e., morphological and fitness attributes), psychosocial (PSWB) attributes also play an important role in police work. How POs approach problems and interact with people can be influenced by their PF and PSWB. Studies suggest that good PF can provide more confidence in verbal approaches, while physical approaches can lead to negative consequences [8]. Indeed, police work is mentally, socially, emotionally, and morally challenging.

In general, the male gender is valued more highly compared to the female gender, and this valuation is observed at the level of family, political, religious, and professional ideologies, from daily tasks and obligations to work and salary benefits [9]. Although the current paradigm is more evolved compared to previous years and there have been numerous advances in the field, society itself still routinely discriminates against the role and position women occupy in everyday life [10]. Despite progress in gender equality, there are still challenges to overcome in accessing certain professions, including the police [11,12]. In recent years, women’s participation in traditionally male-dominated professions has increased significantly. However, there is still a long way to go before women are considered as capable as men at performing operational duties in police agencies [12]. The growing number of women joining the police force in Portugal raises important questions about their PF and PSWB attributes during professional training. The woman travelled and observed greater participation of women in the police force in the 20th century. Understanding the PF status and PSWB attributes of female POs in training is essential to promoting an inclusive, equitable, and healthy work environment.

In Portugal, it is the Internal Security Law that establishes the security forces and services. According to Portuguese Law No. 53/2007 of 31 August, the Public Security Police (PSP) is a “uniformed and armed security force” whose mission is to “ensure democratic legality, guarantee internal security and the rights” of citizens. This force is a highly hierarchical institution that has several tasks that require human resources capable of fulfilling its mission. The PSP includes the National Directorate, the police units (Special Police Unit and territorial police commands), and the two training institutes (Higher Institute of Police Science and Internal Security and Police Officer Academy) to accomplish its tasks.

The Special Police Unit includes (i) the riot sub-unit (Intervention Corps—CI), (ii) the bodyguard sub-unit (Personal Security Corps—CSP), (iii) the special operations and anti-terrorist sub-unit (Special Operations Group—GOE), (iv) the bomb disposal and underground security sub-unit (Centre for Inactivation of Explosives and Underground Security—CIEXSS), and (v) the police dog sub-unit (Canine Technical Operational Group—GOC). Since the creation of the Special Police Unit, several women have attended and completed the courses. However, even if they pass the physical tests, candidates are not always admitted to the final selection phase, which always depends on the number of available positions. Similarly, even if the final selection test is completed, the vacancies are filled with the best candidates. It should be noted that the bodyguard sub-unit has the highest percentage of women eligible to apply (56.44%), and the highest number of women eligible for the sub-unit (note that the data only covers the period from 2010 to 2023). Currently, there are 16 women in the Special Police Unit (10 of whom belong to the bodyguard sub-unit, i.e., six agents, three chiefs, and one officer).

The task entrusted to the bodyguard sub-unit is one of great responsibility, and the POs of this sub-unit is exposed to a high level of physical and psychosocial stress. To cope with such a circumstance, a variety of characteristics and skills are required of the POs who belong to it (e.g., that they are healthy, mentally strong, and physically fit). Hence, PF plays a fundamental role, not only at the time of admission but throughout their careers. Given this backdrop, it becomes imperative to understand the interplay between PF and PSWB attributes, not only essential for their well-being but also for the broader efficacy and inclusivity of the police force.

Following and given the lack of knowledge about the current PF and PSWB profile of women POs, this study aims (i) to identify the PF and PSWB attributes that significantly distinguished women from three professional PO training courses (agents, chiefs, and officers); and (ii) to assess the significance of PF and PSWB attributes on the likelihood that women in professional PO training courses belong to special bodyguard police sub-unit.

## 2. Materials and Methods

### 2.1. Study Design

The study is a cross-sectional analytical study. Based on the objectives of the study and the preparations previously carried out, the planning of the days on which the assessments were to be carried out was agreed with the cadets of the Higher Institute of Police Science and Internal Security (Lisbon, Portugal), the trainees of the training courses for chiefs and agents. This phase corresponds to the data collection (which was conducted in March 2023). Throughout the data collection period, the voluntary system (convenience sample, a non-probability sampling method) was used (volunteer bias), with participants from the professional PO training courses (agents; chiefs; officers) and special bodyguard police sub-unit being informed verbally and in writing of the objectives of their participation and the possibility of declining to participate at any time, which did not occur. Inclusion criteria were the absence of medical contraindications for physical exercise and consent to participate in the study. The research project met the conditions of the Declaration of Helsinki: Recommendations Guiding Physicians in Biomedical Research Involving Human Subjects and had the approval of the Higher Institute of Police Science and Internal Security (reference number: 283/SECDE/2022, 29 November 2022).

### 2.2. Participants

A total of 102 female POs, i.e., 94 female POs from the three professional training courses (agents, n = 48; chiefs, n = 18; officers, n = 28), and 8 from the special bodyguard police sub-unit (Personal Security Corp—CSP) participated in the study.

### 2.3. Physical Fitness Profile

In PF evaluations, were considered in two dimensions, i.e., morphology and fitness.

In accordance, the morphological evaluations included (i) height; (ii) weight, using a bioimpedance scale (OMRON BF511, Omron Inc., Osaka, Japan), which also allows recording the percentage of fat mass (%FM), and the percentage of muscle mass (%MM); (iii) body mass index (BMI); (iv) waist circumference (WC), with the participant standing, arms at the sides of the body, feet together, and abdomen relaxed with the measurement being taken at the narrowest part of the trunk at the level of the navel; (v) hip circumference (HC) with the participant standing and legs slightly apart, with the measurement being taken at the maximum circumference of the hip above the buttocks; (vi) waist-to-hip ratio (WHipR), calculated with the WC and HC [13]; and (vii) waist-to-height ratio (WHR), calculated with the WC and height [13].

The fitness assessment protocol consisted of performing the following fitness tests: (i) push-ups (in 90 s); (ii) sit-ups; (in 120 s); (iii) horizontal jump; and (iv) a 1000 m run.

I. The push-up test aims to assess the muscular resistance strength of the upper body, which is important for situations in which it is necessary to push, and/or apply force [14]. To carry out this test, a space with a flat floor and a stopwatch are needed to administer this test. All participants will begin the test in the starting position, i.e., prone, with upper limbs extended and hands on the floor (shoulder width apart and fingers facing forward). The participant is informed that from the starting position, she must bend her elbows so that her chest is close to the floor (until touching the handle of the counter) and return to the starting position, with full extension of the elbow obligatory. The goal of the test is to perform the described movement as many times as possible within 90 s.
II. The sit-up test is carried out in the context of the importance of abdominal resistance when performing police tasks involving the use of force [14]. To administer the test, all that is needed is a suitable floor, a stopwatch, and a person to count the number of repetitions each participant performs. All participants start the test in the starting position, i.e., supine, with their feet fixed (on the backrest or counter) and resting on the floor. The knees must be bent at approximately 90° and the hands rest on the back of the neck (without going beyond the level of the ears throughout the test). From the starting position, the participant is informed that she must raise her body until her elbows touch the knees and then return to the starting position with shoulder blades touching the floor. The goal of the test is to perform as many repetitions as possible in a maximum time of 120 s.
III. The horizontal jump test aims to measure lower body power and is commonly used with police academy recruits [13]. To perform this test, all that is needed is a tape measure to mark the starting and ending points. Participants begin the test in a standing position. The jump must be performed with both feet at the same time, using the swing of the arms and the flexion of the knees. The distance between the start line of the jump and the landing point is counted, regardless of which part of the body touches the ground. The best score will be recorded (in cm).
IV. For the 1000 m run, participants started in a free position with the body completely behind the starting line. The start signal is given by saying “Wait, wait, now” and from that moment the participants must run the 1000 m in the shortest possible time, recording the elapsed time after crossing the finish line (using a stopwatch). This test is a single repetition. Aerobic capacity was calculated indirectly, considering the time it took the participant to run the 1000 m, i.e., *V*O_2_max = 652.17 − time/6.762 (*V̇*O_2_max, in mL/kg/min; t, in seconds) [15].

The first three tests had the option to be repeated twice.

### 2.4. Psychosocial Profile

To study the PSWB profile of the participants, instruments proposed by Tharion et al. [16] were used, i.e., (i) Grit-S (Short Grit Scale) and (ii) DRS-II M (Dispositional Resilience Scale II Military Version).

The Grit-S was developed by Duckworth and Quinn [17] to measure perseverance and passion for long-term goals. It consists of 8 items divided into two subscales: four items (2, 4, 7, and 8) measure perseverance in effort, and the other four (1, 3, 5, and 6) measure consistency of interest [17]. The items are scored on a scale of 1 to 5, with items 2, 4, 7, and 8 scored in reverse order as follows: questions 2, 4, 7, and 8 (5 = very like me; 4 = mostly like me; 3 = somewhat like me; 2 = not very like me; 1 = not at all like me) and questions 1, 3, 5, and 6 (1 = very like me; 2 = mostly like me; 3 = somewhat like me; 4 = not very like me; 5 = not at all like me). The subscale scores are obtained by calculating the arithmetic mean of the items.

The DRS-II M was used as another method to assess resilience and robustness, having previously been used to assess resilience in a military population [18,19]. It includes three positive subscales (engagement, control, and challenge) and three negative subscales (alienation, powerlessness, and rigidity) divided into 18 questions [18], as shown in Table 1.

The positive dimensions indicate the presence of resources to combat stress, and high scores on these traits are associated with high levels of greater robustness [18]. On the other hand, the negative dimensions indicate greater vulnerability to stress, and low scores on these characteristics indicate high levels of greater robustness [18]. Each statement is rated on a Likert scale ranging from 1 (definitely false) to 5 (definitely true). The subscale scores are obtained by calculating the arithmetic mean of the items.

### 2.5. Statistical Analysis

In general, descriptive statistics was used to characterize the sample, i.e., measures of central tendency (mean, M) and dispersion (standard deviation, SD).

To assess the significance of differences in PF and PSWB attributes among women in the three professional training courses, the Kruskal–Wallis nonparametric test was used, followed by multiple comparisons. In continuation, all attributes were included in the stepwise discriminant analysis (Wilks-Lambda method) to determine which of the attributes examined significantly distinguished women from the three groups of PO training courses (agents; chiefs; officers). The assumptions of normality and homogeneity of the variance-covariance matrices of each group were tested using the Shapiro–Wilk test and Box’s M test, respectively. Although some variables do not have a normal distribution, they were included in the analysis because the observed variance is not very large (in these situations, the discriminant analysis is robust to violations of normality). According to Box’s M test, the assumption of homogeneity of the variance-covariance matrices is also valid (*M* = 55.760; *F*(42.3112.35) = 1.095; *p* = 0.311). Finally, classification analysis was used to obtain classification functions to predict which training group case studies could be classified.

In continuation, to assess the significance of differences in PF and PSWB attributes between females from the professional PO training courses and from the special bodyguard police sub-unit, the nonparametric Mann–Whitney test was used. A type I error probability (alpha) of 0.05 was used. Complementarily, to separately assess the importance of significant attributes on the likelihood of belonging to the special bodyguard police sub-unit, logistic regression with the stepwise forward method was used (LR).

The Statistical Package for the Social Sciences (IBM Corp. Released 2021. IBM SPSS Statistics for Windows, version 28.0. Armonk, NY, USA: IBM Corp.) was used for descriptive and inferential statistical analysis (with *p* ≤ 0.05).

## 3. Results

### 3.1. Professional PO Training Courses

Significant differences were observed between women from the three professional PO training courses for the age ($X_{KW}^{2}$(2) = 37,021, *p* < 0.001, n = 48), morphological (height, $X_{KW}^{2}$(2) = 9952, *p* = 0.007, n = 46; waist-to-hip ratio, $X_{KW}^{2}$(2) = 20,444, *p* < 0.001, n = 46), fitness (horizontal jump, $X_{KW}^{2}$(2) = 16,516, *p* < 0.001, n = 44) and psychosocial (perseverance in effort, $X_{KW}^{2}$(2) = 17,543, *p* < 0.001, n = 47; alienation, $X_{KW}^{2}$(2) = 13,261, *p* = 0.001, n = 48) attributes. Complementarily, multiple comparisons showed that (i) chiefs are significantly older than agents and officers; (ii) officers are significantly taller, have a higher waist-to-hip ratio, are more powerful in the lower limbs (horizontal jump), and have a higher score in the PSWB dimension “perseverance in the effort”, compared to agents and chiefs; and (iii) agents have a significantly higher score on the PSWB dimension “alienation”, compared to chiefs and officers. Results are presented in Table 2.

In continuation, it was observed that stepwise discriminant analysis extracted two discriminant functions, leaving the waist-to-hip ratio, waist-to-height ratio, relative fat mass, relative muscle mass, horizontal jump, and perseverance in the effort as statistically significant attributes, i.e., (i) Function 1 is essentially defined by waist-to-hip ratio and waist-to-height ratio, explaining 61.8% of the variability between groups (lambda = 0.369; *X*^2^(12) = 69.253; *p* < 0.001); and (ii) Function 2 is defined by relative fat mass and relative muscle mass, and significantly discriminates the three professional PO training courses (lambda = 0.667; *X*^2^(5) = 28.149; *p* < 0.001).

The ranking functions are as follows: (i) agents = 1561.686 × WHipR − 3085.662 × WHR + 35.675 × %MG + 47.695 × %MM + 0.898 × HJ + 17.339 × Perseverance in effort − 1275.023; (ii) chiefs = 1542.294 × WHipR − 2968.441 × WHR + 34.488 × %MG + 46.530 × %MM + 0.901 × HJ + 14.804 × Perseverance in effort − 1230.714; and (iii) officers = 1619.947 × WHipR − 3135.458 × WHR + 35.716 × %MG + 47.843 × %MM + 0.980 × HJ + 17.360 × Perseverance in effort − 1316.075.

**Figure 1:**
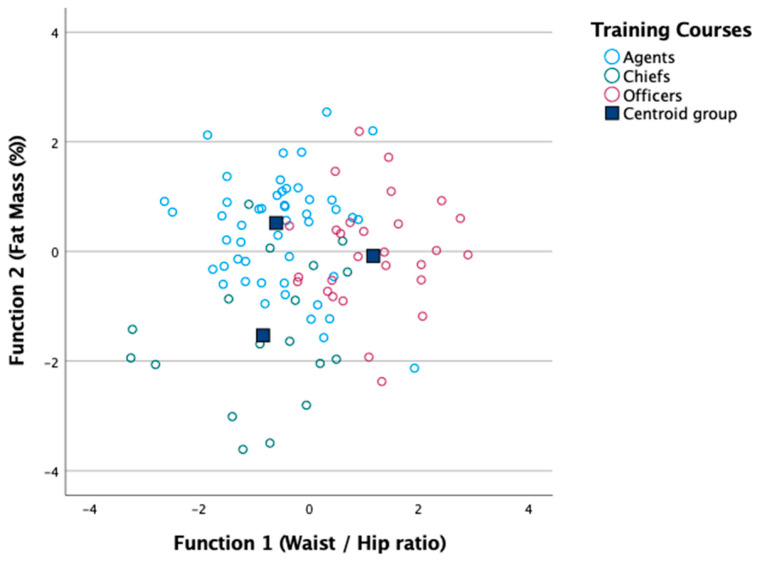
Territorial map of the two discriminant functions.

Table 3 shows the standardized coefficients of these variables in the discriminant functions, the significance of each of these functions, and the percentage of variance between groups explained by the discriminant functions, and Figure 1 shows the positioning of each subject on the territorial map of the scores of the two discriminant functions (with the percentage of individuals classified correctly with the original classification being 72.3%).

In Figure 1 the positioning of each subject on the territorial map of the scores of the two discriminant functions is illustrated, with the percentage of individuals classified correctly with the original classification being 72.3%.

### 3.2. Professional PO Training Courses and Bodyguard Special Police Sub-Unit

Significant differences were found between women in the professional PO training courses and special bodyguard police sub-unit in terms of age (*U* = 712.0, *W* = 748.0, *p* < 0.001), waist-to-hip ratio (*U* = 426.5, *W* = 447.5), and abdominal resistance (sit-ups: *U* = 389.0, *W* = 410.0, *p* = 0.038). The results are shown in Table 4.

When considering the constructs that had attributes with significant differences, it was found that the logistic regressions revealed that the waist-to-hip ratio (morphological model: b_WHipR_ = 16.702, *X*^2^_Wald_ (1) = 3.665, *p* = 0.057) and the resistance abdominal musculature measured using the sit-ups test (fitness model: b_Sit-Ups_ = 0.109, *X*^2^_Wald_ (1) = 4.771, *p* = 0.029) have a statistically significant effect (marginal in the morphological model) on the logit of the probability that women in professional PO training courses belong to the special bodyguard police sub-unit, according to the fitted models (morphological model, *G*^2^(8) = 3.535, *p* = 0.060, *X*^2^_HL_(8) = 12.265; *p* = 0.140, *R*^2^_CS_ = 0.035; *R*^2^_N_ = 0.096; *R*^2^_MF_ = 0.078; fitness model, *G*^2^(1) = 5.882, *p* = 0.015, *X*^2^_HL_(8) = 7.799; *p* = 0.453, *R*^2^_CS_ = 0.070; *R*^2^_N_ = 0.189; *R*^2^_MF_ = 0.156). The probability of belonging to the special bodyguard police sub-unit increases exponentially with the waist-to-hip ratio and sit-ups (11.5% for each repetition). The probability of belonging to the special bodyguard police sub-unit (y = 1) as a function of morphological and fitness profiles can be written probabilistically as follows:

$$\mathrm{Morphological}\;\mathrm{model},\;\mathrm{Logit}\;(\pi )=\frac{1}{1+e^{-\left[-14.876+16.702\;WHipR\right]}}$$

$$\mathrm{Fitness}\;\mathrm{model},\;\mathrm{Logit}\;(\pi )=\frac{1}{1+e^{-\left[-9.183+0.109\;Sit-Ups\right]}}$$

Adjusted logistic regression models were also used to classify the individuals studied, and the percentage of correct classification for both models was ~94% (Table 5).

## 4. Discussion

This study examines the different dimensions of PF, as well as PSWB attributes. To achieve these goals, given the lack of knowledge about the current PF and PSWB profile of female POs, this study comprises two parts that complement each other.

### 4.1. Professional PO Training Courses

Regarding the first part, it was found that in the professional PO training courses, the average (i) agent is 25.94 years old, 164.00 cm tall, and 63.64 kg heavy; (ii) chief is 35.89 years old, 162.28 cm tall, and 60.93 kg heavy; and (iii) officer is 26.00 years old, 166.00 cm tall and 62.97 kg heavy. Of these variables, age stands out as being significantly higher among chiefs.

It was also found that the distribution of PF and PSWB attributes is not the same among women from different professional PO training courses.

In the morphological attributes, the significant difference found in height and waist-to-hip ratio is noticeable. Regarding height, the women in the officers’ training course have a higher average height. However, according to Seidell et al. [20], it is important to note that the waist-to-hip ratio may not be an accurate measure of visceral fat, as hip circumference includes other structures such as bone, gluteal muscle, and gluteal subcutaneous fat. For this reason, the same authors believe that waist circumference may be a more useful measure of abdominal fat.

The women in the officer training course have higher waist-to-hip ratio scores compared to the other participants, which can be attributed to their high waist circumference and lower hip circumference scores. Similarly, they are the ones who have a higher hip circumference (71.59 cm). Although a higher waist circumference may imply a higher level of abdominal fat, in the present case it is not the women who have higher waist circumference values that have higher relative fat mass values. This situation may be explained by the distribution of body fat, which cannot be measured without appropriate equipment. The first discriminant function, defined by waist-to-hip ratio and waist-to-height ratio, explains 61.8% of the differences observed between the three professional POs training groups, while the second discriminant function, defined by relative fat mass and relative muscle mass, explains 38.2%.

A person’s body composition is another method of assessing a person’s PF, often used as an indicator of a person’s overall health and as a predictor of functional ability [21]. Dawes et al. [22] concluded that a higher-than-normal relative fat mass negatively affects POs performance in ground arm extensions, vertical jump, and aerobic capacity compared to POs who have a lower percentage (this is because body fat can increase resistance to oxygen flow and impair muscle oxygen-carrying capacity). Also, the results of studies conducted in elite units [7,23] showed that POs who belong to these units have a lower percentage of fat mass than other colleagues and fellow citizens. Nevertheless, body composition is determined by lifestyle and genetics, and the dimorphism between male and female body composition is evident from an early age and throughout all stages of human development, becoming more apparent in adulthood [21,24]. In general, females have more fat mass and less lean mass than males, mainly due to the effect of sex hormones on each sex [21,24,25].

Also regarding age, Kukić et al. [21] conducted a study with the aim of understanding whether there are significant differences in body composition between female POs of different ages, finding an increase in body mass index values, i.e., as people age, not only women but also men, tend to lose lean mass, resulting in a loss of strength, power, speed, flexibility, and cardiorespiratory capacity [25]. Given the indirect relationship that appears to exist between age and PF, POs must strive to maintain their level of PF as they age [4].

In terms of fitness attributes, it was found that (i) the women in the officers’ training course perform the highest number of repetitions of push-ups and have a superior performance in horizontal jump; (ii) it is the women in the chiefs training course who perform the highest number of sit-ups and run the 1000 m in the shortest time (and as a result of this finding, they have better predicted *V*O_2_max values).

Of the four studied fitness attributes, a very significant difference was found in the horizontal jump. This fitness test evaluates lower limb strength and is related to the leg strength and height of the individual [26], i.e., it seems that leg muscle strength contribution is 9.24% and height contribution is 22.75% [26]. In the situation in question, we found higher average body size and better results in the horizontal jump in females from the officers’ training course.

The cardiorespiratory fitness is also considered an important parameter for police professionals’ health [3]. This consists of the body’s ability to continuously perform the same task through the intake and use of oxygen [2], and oxygen consumption (*V*O_2_max) is one of the most used measures for this criterion [3]. Maupin et al. [3] found that POs who belong to an elite force have a higher relative *V*O_2_max compared to other POs and fellow citizens.

When looking at the PSWB attribute, it is possible to see if there are differences between the three professional PO training courses. The average scores of the GRIT-S reflect that the women in the officers’ training course have higher average Grit scores than the women in the agents’ and chiefs’ training courses. Comparing the three groups, women from the officers’ training course presented higher scores in the “perseverance in the effort”, the difference of which is significant for the average of the agents’ and chiefs’ groups.

A study by Alhadabi and Karpinski [27], which examined the relationship between Grit level and the academic performance of university students, found that the Grit dimension, particularly the “perseverance in the effort”, had a positive impact on students’ autonomy and, consequently, on the effort they invest in their goals and academic performance. In this way, we can reflect on the commitment that the women of the three professional PO training groups show to their current path and their future goals. In accordance, these results suggested that it is the average woman in the officers’ training course who exhibits greater determination and mastery of her goals.

As for DRS, the only variable expressed in the confrontation of scores between the three groups is “alienation”. Nevertheless, since this variable is a negative dimension, the higher the value, the greater the vulnerability to stress. In the present case, the women from the agents’ training course scored higher on average than the women from the other two professional PO training courses. Also, Lovering et al. [19], in a study that examined the physical and mental attributes of military personnel, showed that recruits who scored high in the positive categories of the DRS and low in the negative categories entered the course with greater physical availability and mental resilience. Moreover, other research has shed light on the main stressors faced by POs. Le Scanff and Taugis [28] identified key sources of stress, including PF, organizational situations, leadership, family, work, and media, among special units of the French police. Additionally, Tharion [16] emphasized the significance of traits like grit, resilience, and perseverance in coping with work-related challenges, interpersonal relationships, and social pressures.

Considering the interconnectedness of PF and PSWB, it becomes crucial to address these factors to optimize the performance and overall health of female officers within the Portuguese PSP. The study’s findings showed significant differences in PF attributes across the professional PO training groups, with women in the officers’ course demonstrating higher scores in height and waist-to-hip ratio, and participants in the chiefs’ course excelling in sit-ups and the 1000 m run. Furthermore, PSWB attributes varied, with women in the officers’ course exhibiting more perseverance and commitment to their goals, while women in the agents’ course were more susceptible to stress.

PF is undeniably presented here as a factor closely intertwined with the PSWB of POs. The negative effects of situations such as sleep deprivation, inconsistent eating habits, and night work on POs PF are evident [28]. Building on these findings, further research examining the relationship between PF and PSWB attributes could provide invaluable insights. Understanding how these elements interact and influence each other would provide a more comprehensive understanding of the overall well-being and resilience of female POs in the Portuguese Public Security Police (PSP). Such research could inform the development of holistic support programs that combine targeted physical training with psychosocial support to empower women POs and provide them with the tools to thrive in their demanding roles effectively. By examining the interrelationship between PF and PSWB attributes, we can promote a healthier and more resilient police force, ultimately enhancing Portuguese Public Security Police skills and their ability to serve and protect the community.

### 4.2. Professional PO Training Courses and Bodyguard Special Police Sub-Unit

In the second part of the study, it was found that, in terms of the age and body composition of the participants, the average woman in the professional PO training courses is 29.30 years old, has a height of 164.27 cm, and a body weight of 62.90 kg. On the other hand, the average woman belonging to special bodyguard police sub-unit has an average age of 46.25 years, a height of 164.26 cm, and a body mass of 62.53 kg. The age variable, which shows a very significant difference, can be explained by the difference between the stage of life in which the professional PO training courses are attended (beginning of the professional career) and the stage in which one enters the special bodyguard police sub-unit.

The distribution of morphological attributes is not the same between the studied groups, i.e., it is possible to verify that women from the special bodyguard police sub-unit have a significantly higher waist-to-hip ratio than the women from the professional PO training courses (0.75 vs. 0.71). In general, the average participant in this study has a lower mean waist-to-hip ratio compared with a sample of civilian women (0.79 ± 0.08) in the study by Seidell et al. [20], whose aim was to define the contribution of various measures of body composition to the assessment of fat distribution and the determination of risk factors for cardiovascular disease. In the world of the police, it can be noted that the value obtained in the present study gives better values compared with those obtained in the study of Yates et al. [29] on female POs in operational and administrative functions (0.88 ± 0.20 and 0.80 ± 0.07, respectively). Looking at the absolute values obtained, they show that the average waist circumference of the women in the special bodyguard police sub-unit is higher (73.83 vs. 70.44 cm). Nevertheless, both verified values are within the range indicated by the American College of Sports Medicine (ACSM) for women with a low health risk (70–89 cm) [30].

It was also observed that the participants of the special bodyguard police sub-unit have better values in body mass index (BMI), relative fat mass, and relative muscle mass than the women from the professional PO training courses. This demonstrated that the morphological differences between the two groups do not correspond to the expectations resulting from the comparison carried out by Kukić et al. [21] in female POs of different ages, which showed that there is an increase in fat mass with increasing age. On the other hand, this tendency is supported by Araujo et al. [31], whose study of a Special Police Unit with an average age of 42.6 years shows that it is not possible to attribute the change in individual body composition to age. Since it is not possible to assess whether there was an increase in fat mass as a function of the age of the participants of the special bodyguard police sub-unit, only to note that the relative fat mass is lower compared to women in professional PO training courses (whose average age is lower), we tend to agree with Araujo et al. [31] when they point out that the commitment that these women, belonging to an elite force, have in maintaining their PF may be an explanation for the difference. The assumption of Araujo et al. [31] is supported by Teixeira et al. [32], which shows that in male POs who do not belong to an elite unit, age has a detrimental effect on the morphological attributes.

Regarding BMI, women from the special bodyguard police sub-unit and professional PO training courses have lower values than most of the Portuguese population (23.18 vs. 23.22 vs. 25.8 kg/m^2^, respectively), than an elite group in Portugal aged 28–55 years (23.18 vs. 23.22 vs. 26.6 kg/m^2^, respectively), and similarly aged Portuguese male POs (23.18 vs. 23.22 vs. 27.76 kg/m^2^, respectively) [31,32,33]. According to the Expert Panel on the Identification, Assessment, and Treatment of Overweight and Obesity in Adults, the average BMI of participants is considered normal (18.5–24.9 kg/m^2^) [30]. Nevertheless, a limitation of BMI is that it does not distinguish between fat, muscle, or bone mass, which can lead to errors in body composition assessment (as individuals with a lot of muscle mass can have a BMI > 30 kg/m^2^ and not be obese) [30].

Results showed a significant difference in sit-ups (62.33 vs. 53.01 repetitions), with women from the special bodyguard police sub-unit scoring higher. The scores are also higher in the other tests, except push-ups (it was the women in the professional PO training course who performed more repetitions, i.e., 38.20 vs. 39.49 repetitions). According to Lockie et al. [34], the lower number of push-ups can be justified by the higher waist-to-hip ratio, because the higher the waist-to-hip ratio, the lower the number of repetitions of push-ups.

The protocol for the execution of push-ups and sit-ups used in the special bodyguard police sub-unit, which served as the basis for the study, provides for an execution time of 90 s. There do not appear to be any studies in the literature consulted whose evaluation data on female POs resulted from the use of the same protocol. Complementarily, given the scoring table used in the special bodyguard police sub-unit, and considering the average age of these participants (age, 46.29 years), 63.33 repetitions of sit-ups correspond to 19.50 values (range, 0 to 20), 38.20 push-ups correspond to 20 values (range, 0 to 20), and 4 min and 28 s in the 1000 m correspond to 20 values (range, 0 to 20). For the women in the professional POs training groups, whose average age is 27.86 years, 53.01 repetitions of sit-ups have 12 values (range, 0 to 20), 39.49 push-ups have 14.50 values (range, 0 to 20) and 4 min and 34 s in the 1000 m have 8 values (range, 0 to 20).

There is no consensus in the literature on whether strength is a variable that is negatively affected by age. Although several studies show that there is evidence of a decrease in muscle strength exercises with age, others state that there is no significant difference in women across age groups [22,31]. In accordance, the difference in the performance of the different tests can be attributed to several factors, such as (i) the level of physical condition; (ii) the type of training performed; and (iii) cultural or social factors that influence the level of physical activity [5].

Regarding the 1000 m test, we are not aware of any studies that have used this protocol to evaluate aerobic capacity. Therefore, the values are compared with the classification table of the physical tests at the Special Police Unit and with the cut-off values for professional PO training courses in the Higher Institute of Police Science and Internal Security and Police Officer Academy, i.e., 4 min 35 s, and 5 min, respectively. According to the data, the mean value for both training institutes is above the cut-off value for this test. Complementarily, the prediction of the *V̇*O_2_max over 1000 m shows that the women of the special bodyguard police sub-unit obtained better results than the professional PO training group (56.84 vs. 55.99 mL/kg/min, respectively), although the difference is not significant. Furthermore, the predicted *V̇*O_2_max of the study participants is on average higher than that of elite POs in Portugal aged 29–55 years (50.1 mL/kg/min), and that of Portuguese male POs with a similar average age as the participants (37.1 mL/kg/min) [31,32]. In Riebe et al. [30], (i) 56.84 mL/kg/min (value obtained by women from the special bodyguard police sub-unit) in the 40–49 years age group is in the 95th percentile and is considered higher; and (ii) 55.99 mL/kg/min (value obtained by women from professional PO training courses) in the 20–29 years category is in the 85th percentile and is considered excellent.

In continuation, comparing the GRIT-S test results between the two groups of women in our study, we can see that the average woman from the special bodyguard police sub-unit has higher scores in both categories than the average woman from the professional PO training courses (“persistence in the effort”, 4.29 vs. 4.24; “consistency of interest”, 4.00 vs. 3.89), and according to Duckworth and Quinn [17], all participants scored high on the GRIT-S test, indicating that they are people with high levels of grit. In fact, in Duckworth and Quinn’s [17] study which validated this instrument, responses were analysed by age group. Using the 45–54 years category (since the average age of participants in the special bodyguard police sub-unit is 46.29 years), we find that the women of the special bodyguard police sub-unit have higher scores in both the “persistence in effort” and “consistency of interest” categories than participants in Duckworth and Quinn’s study (4.29 vs. 3.8 and 4.00 vs. 3.0, respectively). Similarly, among participants in professional PO training courses (ages, 25–34 years), both scores are higher than among participants in Duckworth and Quinn’s [17] study (“persistence of effort”, 4.24 vs. 3.6; “consistency of interest”, 3.89 vs. 2.9).

Concerning DRS-II, the present study shows that, compared to the scores of women from the professional PO training courses, the participants from special bodyguard police sub-unit have lower scores in the three positive categories of resilience/robustness and higher scores in the negative categories, except for the measure “stiffness” (2.71 vs. 3.01).

Because there were no significant differences in the PSWB attributes, logistic regression models were created only for the first two constructs (morphological model and fitness model). In accordance, the results of logistic regression showed that waist-to-hip ratio and sit-ups are the attributes that have the largest contribution to the probability of belonging to the special bodyguard police sub-unit. More specifically, the greater the waist-to-hip ratio and number of sit-up repetitions, the greater the probability of belonging to the special bodyguard police sub-unit group (however, this information must be interpreted with some caution given to the reduced sample of special bodyguard police sub-unit participants).

In sum, based on the observed findings, this study highlights (i) the potential benefits of continued monitoring and adaptation of training and support programs (which are crucial for promoting a resilient and effective police force, particularly for female POs in demanding law enforcement roles), and (ii) the practical implications for the Portuguese Public Security Police and other law enforcement agencies, emphasizing the importance of tailoring training programs to address specific fitness attributes and psychosocial aspects for each training course. Nevertheless, to enhance the validity of the study’s findings, it’s essential to acknowledge the limitations associated with the sample size and composition, i.e., that (i) the group sizes (i.e., agents, chiefs, officers) vary significantly, and this imbalance can affect the statistical analyses and the validity of group comparisons (balance across groups); (ii) the relatively small sample sizes for each group, conducting subgroup analyses (e.g., comparing PF or PSWB attributes within each training course) might be limited in statistical power (potential for subgroup analysis); and (iii) only eight participants from the special bodyguard police subunit is a very small sample size, and it may be challenging to draw robust conclusions about this group (the special bodyguard police sub-unit). Considering the limitations stated, it seems important to highlight that the results can be interpreted as indicative of trends or patterns within this specific sample rather than making broad generalizations about all female police officers.

## 5. Conclusions

The daily need for POs to do their job makes it imperative that they find a balance, not only between motor and functional PF but also with the personal and social relationships that come with the job to ensure that both are positive.

Given the results obtained, we can conclude, about the professional PO training courses, that: (i) there is a significant age difference between the women in the chiefs training course and the rest of the groups (but this does not affect the PF); (ii) the women who participate in the agents training course are the ones who, on average, perform the worst in the fitness tests; and (iii) the women in officers’ training course score higher overall in the PSWB tests, which translates into higher levels of drive and robustness.

Regarding the PF and PSWB profiles of the women in the professional PO training courses for agents, chiefs, and officers, differences were found compared to the women in the special bodyguard police sub-unit. Although the women from the special bodyguard police sub-unit have a higher average age, they are in better physical condition and have a higher level of demands than the other evaluated women. At the PSWB level, there were no significant differences. In short, PF distinguishes who belongs to an elite unit and who does not.

This study serves as a valuable resource for the Portuguese Public Security Police and other law enforcement agencies seeking to improve the PF and PSWB of female officers. The findings can be used to design targeted training programs that address specific fitness attributes and PSWB aspects for each training course. In addition, the results can inform recruitment strategies and age-specific fitness guidelines. Further research can build on this study by examining the long-term effects of PF and PSWB attributes on job performance, job satisfaction, and overall health of female PO. Continued monitoring and adaptation of training and support programs based on these findings can help promote a resilient and effective police force that supports the well-being and effectiveness of female POs in challenging law enforcement roles.

## Figures and Tables

**Table 1:** Distribution of questions by DRS-II M subscales.

Subscales		Questions
Positive	Control	1, 7, 13
	Commitment	3, 9, 15
	Challenge	5, 11, 17
Negative	Impotence	2, 8, 14
	Alienation	4, 10, 16
	Stiffness	6, 12, 18

**Table 2:** Age, morphological, fitness, and psychosocial attributes of women in professional police officers (POs) training courses (agents; chiefs; officers).

Variables	Professional POs Training Courses									Statistical Analysis				
	Agents (1)			Chiefs (2)			Officers (3)			KW Test		Multiple Comparation		
	n	Mean	SD	n	Mean	SD	n	Mean	SD	d.f.(2)	*p*-Value	1-2	1-3	2-3
Age (years)	48	25.94	4.12	18	35.89	4.19	28	26.00	5.24	37.021	<0.001	<0.001		<0.001
Height (cm)	46	164.00	4.37	18	162.28	3.61	28	166.00	4.74	9.952	0.007		0.024	0.002
Weight (kg)	48	63.64	7.57	18	60.93	6.96	28	62.97	6.40	1.622	0.444			
BMI (kg/m^2^)	46	23.54	2.30	18	22.93	2.32	28	22.86	2.17	1.837	0.399			
Waist circumference (cm)	46	69.60	5.42	18	70.81	6.26	28	71.59	5.81	3.391	0.184			
Hip circumference (cm)	46	100.15	4.79	18	99.58	6.51	28	97.14	4.91	4.495	0.106			
Waist/Hip ratio	46	0.69	0.04	18	0.71	0.05	28	0.74	0.04	20.444	<0.001		<0.001	0.024
Waist/Height ratio	46	0.42	0.03	18	0.44	0.04	28	0.43	0.03	2.328	0.312			
Fat mass (%)	48	32.68	4.83	18	29.71	5.46	28	30.38	5.24	5.479	0.065			
Muscular mass (%)	48	28.83	2.40	18	30.29	3.21	28	30.15	3.35	5.187	0.075			
Push-ups 90 s (repetitions)	42	37.62	10.62	14	40.07	14.99	28	42.00	10.60	3.594	0.166			
Sit-ups 120 s (repetitions)	43	51.79	9.62	15	57.67	11.42	28	52.39	11.01	3.282	0.194			
Horizontal jump (cm)	44	175.47	13.81	14	177.07	16.58	28	188.96	11.27	16.516	<0.001		<0.001	0.037
1000 m (s)	39	273.90	22.73	12	266.67	30.50	27	276.22	22.20	1.029	0.598			
Predicted *V̇*O_2_max (mL/kg/min)	39	55.94	3.36	12	57.01	4.51	27	55.60	3.28	1.029	0.598			
Perseverance in effort (GRIT1)	47	4.22	0.52	18	3.82	0.71	28	4.54	0.38	17.543	<0.001		0.007	<0.001
Consistency of interest (GRIT2)	47	3.92	0.39	18	3.78	0.48	28	3.91	0.50	0.713	0.700			
Control (DRS1)	48	4.53	0.33	18	4.20	0.92	28	4.32	0.38	5.004	0.082			
Commitment (DRS2)	48	4.11	0.46	18	4.15	0.93	28	4.19	0.56	1.591	0.451			
Challenge (DRS3)	48	3.66	0.59	18	3.83	0.84	28	3.75	0.63	1.703	0.427			
Impotence (DRS4)	48	1.64	0.51	18	1.63	0.69	28	1.40	0.45	3.888	0.143			
Alienation (DRS5)	48	1.89	0.61	18	1.48	0.89	28	1.55	0.46	13.261	0.001	<0.001	0.025	
Stiffness (DRS6)	48	2.98	0.76	18	3.17	0.55	28	2.98	0.79	1.154	0.562			

**Table 3:** Standardized coefficients of the discriminant power variables, the percentage of variance between groups explained by the two extracted discriminant functions, and the significance of the discriminant functions (*p*-value).

Variables	Coefficients in Discriminant Functions	
	1	2
Waist/hip ratio (WHipR)	1.427	0.222
Waist/height ratio (WHR)	−1.573	−1.783
Percent fat mass (%FM)	1.091	2.855
Percent muscle mass (%MM)	0.797	1.608
Horizontal Jump (HJ)	0.600	−0.092
Perseverance in effort (GRIT1)	0.211	0.605
Eigenvalue	0.807	0.499
Explained variance	61.8%	38.2%
*p*-value	<0.001	<0.001

**Table 4:** Descriptive analysis of age, morphological, fitness, and psychosocial attributes of women in professional PO training courses (agents, chiefs, and officers), in the special bodyguard police sub-unit, and Mann–Whitney test statistics.

Variables	Policewomen						Mann–Whitney Test		
	Professional PO Training Courses			Bodyguard Special Sub-Unit					
	n	Mean	SD	n	Mean	SD	U	W	*p*-Value
Age (years)	94	27.86	5.93	8	46.25	10.50	712.0	748.0	<0.001
Height (cm)	92	164.27	4.50	7	164.26	4.96	323.0	351.0	0.989
Weight (kg)	94	62.92	7.12	7	62.53	5.10	336.5	364.5	0.920
BMI (kg/m^2^)	92	23.22	2.27	7	23.18	1.74	328.5	356.5	0.929
Waist circumference (cm)	92	70.44	5.71	6	73.83	5.18	390.5	411.5	0.089
Hip circumference (cm)	92	99.13	5.31	6	98.83	4.12	270.0	291.0	0.929
Waist/Hip Ratio	92	0.71	0.04	6	0.75	0.03	426.5	447.5	0.026
Waist/Height Ratio	92	0.43	0.03	6	0.45	0.04	366.0	387.0	0.182
Fat mass (%)	94	31.42	5.19	6	28.88	5.05	186.5	207.5	0.166
Muscle Mass (%)	94	29.50	2.92	6	30.62	3.65	347.5	368.5	0.342
Push-Ups 90 s (repetitions)	84	39.49	11.46	5	38.20	8.79	195.5	210.5	0.796
Sit-Ups 120 s (repetitions)	86	53.01	10.51	6	62.33	9.61	389.0	410.0	0.038
1000 m (s)	78	273.59	23.76	6	267.83	32.92	210.0	231.0	0.677
Predicted *V̇*O_2_max (mL/kg/min)	78	55.99	3.51	6	56.84	4.87	258.0	279.0	0.677
Perseverance in effort (Grit1)	93	4.24	0.58	7	4.29	0.39	318.5	346.5	0.924
Consistency of interest (Grit2)	93	3.89	0.44	7	4.00	0.43	375.5	403.5	0.490
Control (DRS1)	94	4.41	0.52	7	4.00	1.28	289.5	317.5	0.587
Commitment (DRS2)	94	4.14	0.60	7	3.90	1.20	338.5	366.5	0.897
Challenge (DRS3)	94	3.72	0.65	7	3.67	0.67	316.5	344.5	0.866
Impotence (DRS4)	94	1.57	0.54	7	2.24	1.12	456.5	484.5	0.081
Alienation (DRS5)	94	1.71	0.65	7	2.00	1.09	360.5	388.5	0.668
Stiffness (DRS6)	94	3.01	0.73	7	2.71	1.04	239.0	267.0	0.224

**Table 5:** Synthesis of models adjusted for morphological and fitness profiles.

Model	Improvement			Model			Correct Classification (%)	Variable
	*X* ^2^	d.f.	Sig.	*X* ^2^	d.f.	Sig.		
Morphology	3535	1	0.060	3.535	1	0.060	93.9	IN: waist-to-hip ratio
Fitness	5.882	1	0.015	5.882	1	0.015	93.8	IN: sit-ups (repetitions)

## Data Availability

The data presented in this study are available upon reasonable request from the corresponding author. The data are not publicly available due to privacy and ethical restrictions.
